# Epidermal growth factor receptor intron-1 CA repeat polymorphism on protein expression and clinical outcome in Taiwanese oral squamous cell carcinoma

**DOI:** 10.1038/s41598-017-04954-5

**Published:** 2017-07-10

**Authors:** Shiang-Fu Huang, Huei-Tzu Chien, Wen-Yu Chuang, Chih-Hsiung Lai, Sou-De Cheng, Chun-Ta Liao, Hung-Ming Wang

**Affiliations:** 1 Department of Otolaryngology, Head and Neck Surgery, Chang Gung Memorial Hospital, Tao-Yuan, Taiwan; 2 Department of Public Health, College of Medicine, Chang Gung University, Tao-Yuan, Taiwan; 3 Department of Pathology, Chang Gung Memorial Hospital, Tao-Yuan, Taiwan; 4 Department of Anatomy, College of Medicine, Chang Gung University, Tao-Yuan, Taiwan; 5 Division of Hematology/Oncology, Department of Internal Medicine, Chang Gung Memorial Hospital, Tao-Yuan, Taiwan

## Abstract

This study was designed to explore the relationship between epidermal growth factor receptor (EGFR) CA repeats polymorphism and protein expression in oral cavity squamous cell carcinoma (OSCC). A total of 194 OSCCs were examined for EGFR protein overexpression, gene copy number and the length of their CA repeats. The length of the *EGFR* CA repeats was found not to be associated with *EGFR* gene copy number or with protein overexpression. To exclude the effect of *EGFR* gene copy number on protein overexpression, only those OSCC tumors with disomy of the *EGFR* gene were included in further analysis. In this subgroup, EGFR protein overexpression was significantly associated with poor differentiation of the tumor cells and lymph node metastasis, especially extra-capsular spread. However, *EGFR* CA repeats were not related to any clinicopathological factor. Interestingly, patients genetically found to have the *EGFR* CA repeats SS genotype and having tumors with EGFR protein overexpression were found to have a worst prognosis in terms of disease-free survival (DFS) (HR = 2.68; 95% CI, 1.03–6.98) after multivariate adjustment. The present study demonstrates that concurrent overexpression of EGFR protein in the presence genetically of the SS form CA repeats acts as a predictor for poor DFS.

## Introduction

In Taiwan, oral cancer (including sub-sites in the oral cavity, oropharynx and hypopharynx) is the fourth most common cancer in men^1^. The primary treatment for oral cavity squamous cell carcinoma (OSCC) is radical surgery with or without post-operative chemoradiation^2^. However, for inoperable/recurrent disease or metastasis at distant sites, the patients’ treatment options are limited and their prognosis is usually poor. Recent findings have indicated that epidermal growth factor receptor (EGFR) and its signaling transduction pathway play an important role in head and neck cancer in Taiwan, including areca quid (AQ) associated OSCC^3^. Overexpression of EGFR has been confirmed to occur in AQ associated OSCC and has been reported to be associated with poor prognosis^3–5^. Treatment with an anti-EGFR agent has been reported to improve outcome compared to radiotherapy alone in head and neck cancers^6^. However, the levels of EGFR protein expression were found not to be consistently correlated with treatment response.

EGFR protein overexpression has primarily been attributed to increased transcriptional activity as well as to increases in *EGFR* copy number^7^. Basal transcription of the *EGFR* gene is regulated by Sp1 transcription factor; in this context the CA repeat genotype of intron 1 (rs 11568315) has been shown to contribute to different levels of transcriptional activity^8, 9^. Etienne-Grimaldi *et al*.^9^ have reported that the number of CA repeats is inversely correlated with protein expression in human tumors, including head and neck cancer. However, they were unable to confirm that the number of CA repeats had a significant influence on EGFR expression in a later study^10^. This contradictory result may be mainly due to the complexity of head and neck cancer, which is composed of cancers from a number of different anatomical sites. Thus, whether there is a relationship between the intron 1 CA repeat genotype and protein expression in head and neck cancer is still unresolved.

OSCC is the major head and neck cancer in Taiwan and mechanisms regulating levels of EGFR protein expression in OSCC are not fully understood. We have previously shown that *EGFR* genetic mutations play a very minor role in OSCCs, whereas gene copy number was found to be significantly correlated with EGFR protein overexpression^4^. However, the role of the patient’s *EGFR* intron 1 CA repeat genotype in OSCC is rarely explored^11^. Since the *EGFR* intron 1 CA repeat genotype is known to be associated with the gene’s transcriptional activity, the CA repeat genotype has been implicated in cancer risk and in patient clinical outcome^12^. In this study, we comprehensively investigated the effects of *EGFR* CA repeat genotype on OSCC risk and protein overexpression, as well as evaluating its prognostic role.

## Methods and Materials

### Patients, tissue specimens and clinical diagnosis

This study was approved by the Institutional Review Board, Chang Gung Medical Foundation. The committee approved the experiments, and the informed consent was obtained from all subjects. The methods in this study were carried out in accordance with the relevant guidelines, including any relevant details. A total of 194 male OSCC patients who received primary radical surgery treatment at Chang Gung Memorial Hospital, Lin-Kuo during the period from March 1997 to June 2004 were recruited to participate in the study. All cases gave written informed consent for participation before surgery and all cases were confirmed by histology. For each case, 10 ml of venous blood was drawn and then separated into plasma, buffy coat cells and red blood cells by centrifugation within 18 h of obtaining the blood; the buffy coat cells were then stored at −80 °C. Genomic DNA for *EGFR* intron 1 CA repeats genotyping was purified from the buffy coat cells as described previously^13^. As referent controls, 1444 Taiwanese random males, whose blood was originally collected to study their blood lead concentrations, were also included in this study^14^.

### Fluorescence *in situ* hybridization (FISH) assay to assess EGFR gene copy number

*EGFR* gene copies were investigated by FISH using the LSI *EGFR* SpectrumOrange/CEP 7 SpectrumGreen probe system (Vysis; Abbott Laboratories, Downers Grove, IL) as described previously^4^. At least 100 non-overlapping nuclei per case were scored independently by two independent observers. The FISH patterns were classified into three levels based on the copy number of *EGFR* genes per cell as described in previous studies^4, 15, 16^. These were normal disomy, with ≤two copies in more than 90% of the analyzed cells; low amplification/polysomy (LA/Poly), ≥three copies in more than 40% of the analyzed cells, and gene amplification, which was defined by the presence of tight *EGFR* gene clusters in ≥10% of the analyzed cells.

### Immunohistochemical Analysis of EGFR protein overexpression

Immunohistochemical staining for EGFR protein was processed using anti-EGFR monoclonal antibody NCL-EGFR-384 (1:100) (Novocastra, Newcastle, UK) as described previously^17^. Normal skin, known to be EGFR positive, served as both positive (primary antibody added) and negative (no primary antibody) controls. The specimens were examined for the extent and intensity of nuclear and non-nuclear staining by the pathologist (W.-Y.C.) in a blind manner and scored according to the following criteria: 0, no discernible staining or background type staining; 1+, equivocal discontinuous membrane staining; 2+, unequivocal membrane staining with moderate intensity; and 3+, strong and complete plasma membrane staining. In the present study, when more than 25% of the cells had EGFR membrane staining with intensity scores of 2+ and 3+, then there was considered to be EGFR overexpression^15, 17, 18^.

### EGFR intron 1 CA repeats genotyping

The procedure for analysis of the *EGFR* intron 1 CA repeats length polymorphism was modified from previous reports^11, 19, 20^. Briefly, fluorescein-labeled forward primer 5′-FAM-GTTTGAAGAATTTGAGCCAACC-3′ and reverse primer 5′-GTCTGCACACTTGGCACACT-3′ was used for the PCR reaction, which began with initial heating for 12 min at 95 °C, followed by 30 cycles of denaturation at 94 °C for 30 s, annealing at 60 °C for 60 s, and extension at 72 °C for 60 s. The fragment length of the amplified PCR products based on the 500 LIZ size standards was determined using the ABI Prism 3100 DNA Analyzer with GeneScan software (Applied Biosystems, Foster City, CA). According to the NCBI Build 36.1 reference sequence, the PCR product is predicted to be 116 bp with 16 CA repeats. Homozygous samples were randomly selected for direct sequencing to verify CA repeat number and also used as the internal control for the GeneScan analysis. The primers used for direct sequencing of the CA repeat number were: forward primer 5′-AGAGCTCATCCTGGCCAAC-3′ and reverse primer 5′-GCTCAAGGTTGGAATTGTGC-3′.

### Statistical analysis

Statistical analyses were performed using the SPSS statistical package (SPSS, Chicago, IL). The correlations between the *EGFR* intron 1 CA repeat genotype and age, cigarette smoking, alcohol drinking, AQ chewing, EGFR protein overexpression and clinicopathological parameters was examined by χ^2^ test or Fisher’s exact test as appropriate. Survival curves were constructed by the Kaplan-Meier method and the curves were compared using the log-rank test. The Cox regression model was applied to adjust simultaneously for all potential prognostic variables, including age and lymph node metastasis. A two-sided value of *p* < 0.05 was considered statistically significant.

## Results

### EGFR intron 1 dinucleotide CA repeats polymorphism and OSCC risk

We studied a total of 194 OSCC patients and 1444 referent control individuals (Supplementary Table 1). Twelve different alleles of the CA repeat length within the range of 10 to 24 were observed. The most common allele in both referent controls and OSCC patients was 20 followed by 16 and 15 CA repeats. As illustrated in Supplementary Figure 1A, the allelic distribution in referent controls and OSCC patients were similar. The most common genotype in referent controls were 20/20 (26.45%, 382/1444), 16/20 (20.01%, 289/1444) and 15/20 (9.56%, 138/1444); while the most common genotypes in OSCC cases were 20/20 (26.80%, 52/194), 16/20 (21.13%, 41/194) and 19/20 (7.73%, 15/194) (Supplementary Figure 1B). The distribution of CA repeat genotypes was not significantly different between the OSCC patients and the referent controls (*p* = 0.09).

To assess the association between *EGFR* intron 1 polymorphism and OSCC risk, the number (range: 10–24) of CA repeats in each allele was categorized at the sample median (20). The categories were CA repeat <20, which was named the short (S) form and CA repeat ≥20 which was named the long (L) form. The SS genotype in general was found to be slightly associated with an increased OSCC risk (odds ratio (OR) = 1.40; 95% confidence interval (CI), 0.95–2.05; *p* = 0.08). When stratified by the major risk factors of OSCC, the SS genotype was significantly associated with an increased OSCC risk among AQ chewers (OR = 1.70; 95% CI, 1.04–2.76; *p* = 0.03) (Table 1). Since the mean age of the OSCC patients was 49.28 (standard deviation (SD) = 11.34) years old and that of the referent controls was 46.04 (SD = 16.68), we used an unconditional multivariate logistic regression to adjust this potential confounding variable (age). Individuals with SS genotype were still found to have a significantly higher OSCC risk than those with either the LL or LS genotype (OR = 1.65; 95% CI, 1.01–2.70; *p* = 0.05), especially among AQ chewers (Table 1).

**Table 1:** Associations between *EGFR* CA repeat genotype and OSCC risk.

	CA repeat genotype	Referent controls (n = 1444)	OSCC patients (n = 194)	*p* value	Odds ratio (95% CI)	Odds ratio adjusted for age† (95% CI)
Total subjects	LL form	528 (36.6)	68 (35.1)	0.22	1	1
	SL form	702 (48.6)	88 (45.4)		0.97 (0.70–1.36)	0.98 (0.70–1.37)
	SS form	214 (14.8)	38 (19.6)		1.38 (0.90–2.11)	1.38 (0.90–2.11)
Total subjects	SL + LL form	1230 (85.2)	156 (80.4)	0.08	1	1
	SS form	214 (14.8)	38 (19.6)		1.40 (0.95–2.05)	**1**.**65** (**1**.**01–2**.**70**)
Cigarette smoking						
Yes	SL + LL form	705 (84.9)	139 (79.4)	0.07	1	1
	SS form	125 (15.1)	36 (20.6)		1.46 (0.97–2.21)	1.46 (0.97–2.21)
No	SL + LL form	525 (85.5)	17 (89.5)	1.00*	1	1
	SS form	89 (14.5)	2 (10.5)		0.69 (0.16–3.06)	0.61 (0.14–2.69)
Alcohol drinking						
Yes	SL + LL form	303 (85.6)	107 (81.1)	0.22	1	1
	SS form	51 (14.4)	25 (18.9)		1.39 (0.82–2.35)	1.39 (0.81–2.34)
No	SL + LL form	927 (85.0)	49 (79.0)	0.20	1	1
	SS form	163 (15.0)	13 (21.0)		1.51 (0.80–2.84)	1.50 (0.80–2.83)
AQ chewing						
Yes	SL + LL form	274 (86.7)	139 (79.4)	**0**.**04**	1	1
	SS form	42 (13.3)	36 (20.6)		**1**.**69** (**1**.**04–2**.**76**)	**1**.**65** (**1**.**01–2**.**70**)
No	SL + LL form	956 (84.8)	17 (89.5)	0.76*	1	1
	SS form	172 (15.2)	2 (10.5)		0.65 (0.15–2.86)	0.62 (0.14–2.70)

*Fisher’s exact test. †age dichotomized at 50 years old.

### EGFR protein overexpression, the genotype of the CA repeats and OSCC clinicopathological factors

The genotype of the *EGFR* CA repeats of the OSCC tumors was found not to be associated with gains in the copy number (both low amplification/polysomy and amplification) of *EGFR* gene or with protein overexpression (Table 2). As reported previously^3, 4^, there was a significant association between a gain of *EGFR* gene copy number and protein overexpression in Taiwanese OSCC tumors (data not shown). To rule out the effect on protein overexpression of this increase in copy number of the *EGFR* gene, only those OSCC tumors with disomy of the *EGFR* gene were included in the further analysis.

**Table 2:** The relationship between *EGFR* CA repeat genotype, copy number and protein overexpression.

	CA repeat genotype				
	SS form N (%)	SL form N (%)	LL form N (%)	SL/LL form N (%)	*p* value*
EGFR copy number					
Disomy	29 (76.3)	62 (70.5)	44 (64.7)	106 (67.9)	0.60
Trisomy/polysomy	3 (7.9)	10 (11.4)	8 (11.8)	18 (11.5)	
Amplification	6 (15.8)	16 (18.2)	16 (23.5)	32 (20.5)	
EGFR overexpression					
No	22 (57.9)	40 (45.5)	31 (45.6)	71 (45.5)	0.17
Yes	16 (42.1)	48 (54.5)	37 (54.4)	85 (54.5)	

*Chi-square test comparing SS form and SL/LL form.

In this subgroup, EGFR protein overexpression was found to be significantly associated with poor differentiation of the tumor cells (*p* = 0.003) and lymph node metastasis, especially extra-capsular spread (ECS) (*p* = 0.03) (Table 3). On the other hand, the tumor aggressiveness factors, including bone, skin invasion and perineural invasion were not related to EGFR protein overexpression (Table 3). Interestingly, OSCC patients without a history of alcohol drinking showed a higher frequency of EGFR protein overexpression than those who were alcohol drinkers. However, EGFR protein overexpression was not associated with either cigarette smoking or AQ chewing (Table 3).

**Table 3:** The associations between EGFR protein overexpression, *EGFR* CA repeat genotype and clinicopathological parameters among EGFR disomy OSCC patients (n = 135).

	EGFR protein overexpression		*p* value	CA repeat genotype				*p* value†
	No	Yes		SS form	SL form	LL form	SL + LL form	
Age								
<50 yrs (n = 69)	41 (59.4)	28 (40.6)	0.21	14 (20.3)	32 (46.4)	23(33.3)	55 (79.7)	0.73
≥50 yrs (n = 66)	46 (69.7)	20 (30.3)		15 (22.7)	30 (45.5)	21 (31.8)	51 (77.3)	
Tumor stage								
Early (n = 45)	29 (64.4)	16 (35.6)	1.00	7 (15.6)	26 (57.8)	12 (26.7)	38 (84.4)	0.24
Advanced (n = 90)	58 (64.4)	32 (35.6)		22 (24.4)	36 (40.0)	32 (35.6)	68 (75.6)	
Primary tumor								
T1/T2 (n = 74)	45 (60.8)	29 (39.2)	0.33	14 (18.9)	40 (54.1)	20 (27.0)	60 (81.1)	0.43
T3/T4 (n = 61)	42 (68.9)	19 (31.1)		15 (24.6)	22 (36.1)	24 (39.3)	46 (75.4)	
Differentiation								
Well (n = 68)	52 (76.5)	16 (23.5)	**0**.**003**	13 (19.1)	31 (45.6)	24 (35.3)	55 (80.9)	0.50
Moderate/poor (n = 67)	35 (52.2)	32 (47.8)		16 (23.9)	31 (46.3)	20 (29.9)	51 (76.1)	
Tumor depth								
<10 mm (n = 62)	37 (59.7)	25 (40.3)	0.29	9 (14.5)	32 (51.6)	21 (33.9)	53 (85.5)	0.07
≥10 mm (n = 73)	50 (68.5)	23 (31.5)		20 (27.4)	30 (41.1)	23 (31.5)	53 (72.6)	
Lymph node metastasis								
LN (−); ECS^‡^ (−) (n = 79)	53 (67.1)	26 (32.9)	**0**.**05**	15 (19.0)	41 (51.9)	23 (29.1)	64 (81.0)	0.44
LN (+); ECS (−) (n = 26)	20 (76.9)	6 (23.1)		5 (19.2)	12 (46.2)	9 (34.6)	21 (80.8)	
LN (+); ECS (+) (n = 30)	14 (46.7)	16 (53.3)		9 (30.0)	9 (30.0)	12 (40.0)	21 (70.0)	
Skin invasion								
Yes (n = 15)	13 (86.7)	2 (13.3)	0.06	3 (20.0)	5 (33.3)	7 (46.7)	12 (80.0)	1.00
No (n = 120)	74 (61.7)	46 (38.3)		26 (21.7)	57 (47.5)	37 (30.8)	94 (78.3)	
Bone invasion								
Yes (n = 26)	18 (69.2)	8 (30.8)	0.57	7 (26.9)	11 (42.3)	8 (30.8)	19 (73.1)	0.45
No (n = 109)	69 (63.3)	40 (36.7)		22 (20.2)	51 (46.8)	36 (33.0)	87 (79.8)	
Perineural invasion								
Yes (n = 34)	22 (64.7)	12 (35.3)	0.97	7 (20.6)	11 (32.4)	16 (47.1)	27 (79.4)	0.88
No (n = 101)	65 (64.4)	36 (35.6)		22 (21.8)	51 (50.5)	28 (27.7)	79 (78.2)	
Cigarette smoking								
Yes (n = 120)	79 (65.8)	41 (34.2)	0.34	28 (23.3)	53 (44.2)	39 (32.5)	92 (76.7)	0.19*
No (n = 15)	8 (53.3)	7 (46.7)		1 (6.7)	9 (60.0)	5 (33.3)	14 (93.3)	
Alcohol drinking								
Yes (n = 88)	62 (70.5)	26 (29.5)	**0**.**05**	19 (21.6)	39 (44.3)	30 (34.1)	69 (78.4)	0.97
No (n = 47)	25 (53.2)	22 (46.8)		10 (21.3)	23 (48.9)	14 (29.8)	37 (78.7)	
AQ chewing								
Yes (n = 121)	77 (63.6)	44 (36.4)	0.77*	29 (24.0)	53 (43.8)	39 (32.2)	92 (76.0)	**0**.04*
No (n = 14)	10 (71.4)	4 (28.6)		0 (0.0)	9 (64.3)	5 (35.7)	14 (100.0)	

Abbreviations: LN: lymph node metastasis; ECS: extra-capsular spread; AQ: areca quid. ^*^Fisher’s exact test. ^†^Chi-square test comparing SS form and SL/LL form.

The patient’s *EGFR* CA repeat genotype was found not to be associated with tumor stage, tumor differentiation, lymph node metastasis or tumor aggressiveness factors, including skin, bone and perineural invasion (Table 3). Interestingly, AQ chewing, but not cigarette smoking or alcohol drinking, was significantly associated with the *EGFR* CA repeat genotype. The OSCC patients with the SS genotype were all AQ chewers (Table 3).

### The prognostic implications of the EGFR CA repeat genotype and protein overexpression among OSCC patients with disomy of the EGFR gene

**Figure 1:**
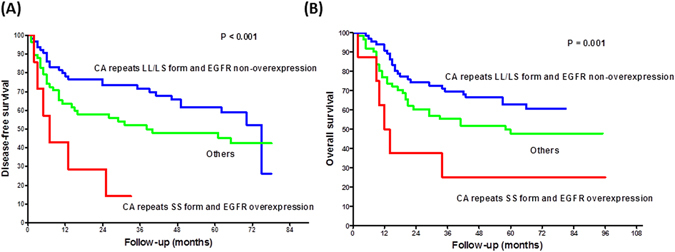
Kaplan-Meier analysis of the combined effect of the *EGFR* CA repeat genotype and protein overexpression on disease-free survival (**A**) and overall survival (**B**) of 135 Taiwanese male OSCCs with disomy of the EGFR gene.

Using univariate analysis, EGFR protein overexpression was slightly associated with disease free survival (DFS) (*p* = 0.07; hazard ratio (HR) = 1.59; 95% CI, 0.97–2.62) and overall survival (OS) (*p* = 0.07; HR = 1.60; 95% CI, 0.97–2.65) (Table 4). Patients with the *EGFR* CA repeat SS genotype had a worse DFS (*p* = 0.09; HR = 1.70; 95% CI, 0.92–3.13) and a worse OS (*p* = 0.03; HR = 1.92; 95% CI, 1.07–3.43). Furthermore, patients found genetically to have the *EGFR* CA repeat SS genotype and a tumor with EGFR protein overexpression had the worst prognosis in terms of both DFS (*p* = 0.002; HR = 4.11; 95% CI, 1.66–10.14) and OS (*p* = 0.01; HR = 3.25; 95% CI, 1.33–7.95) compared to those with either form of the L allele CA repeat genotype and/or no EGFR protein overexpression by their tumor (Table 4, Fig. 1). After multivariate adjustment for age, primary tumor status, lymph node metastasis, tumor depth, and tumor cell differentiation, this significance relationship was still existed for DFS (*p* = 0.04; HR = 2.68; 95% CI, 1.03–6.98) but not for OS (*p* = 0.07; HR = 2.41; 95% CI, 0.95–6.15) (Table 5).

**Table 4:** Univariate analysis of the prognostic covariates for *EGFR* disomy OSCC patients (n = 135).

	Patient no.	Disease-free survival	*p* value	Overall survival	*p* value
		HR (95% CI)		HR (95% CI)	
Age					
<50 years	69	1		1	
≥50 years	66	1.11 (0.68–1.81)	0.68	1.42 (0.86–2.34)	0.17
Primary tumor status					
T1/T2	74	1		1	
T3/T4	61	1.04 (0.63–1.72)	0.88	1.69 (1.03–2.79)	**0**.**04**
Nodal status			**<0**.**001**		**<0**.**001**
(−)metastasis, (−)ECS	79	1		1	
(+)metastasis, (−)ECS	26	0.94 (0.43–2.07)	0.89	1.58 (0.78–3.21)	0.20
(+)metastasis, (+)ECS	30	4.26 (2.47–7.37)	**<0**.**001**	4.21 (2.40–7.36)	**<0**.**001**
Differentiation					
Well	68	1		1	
Moderate/Poor	67	1.08 (0.66–1.76)	0.77	1.24 (0.75–2.03)	0.41
Tumor stage					
Stage I/II	45	1		1	
Stage III/IV	90	2.16 (1.22–3.82)	**0**.**008**	3.30 (1.72–6.37)	**<0**.**001**
Tumor depth ≥10mm					
No	62	1		1	
Yes	73	2.26 (1.35–3.79)	**0**.**002**	2.85 (1.64–4.94)	**<0**.**001**
EGFR protein overexpression					
No	87	1		1	
Yes	48	1.59 (0.97–2.62)	0.07	1.60 (0.97–2.65)	0.07
EGFR CA dinucleotide repeats					
SL/LL form	106	1		1	
SS form	29	1.70 (0.92–3.13)	0.09	1.92 (1.07–3.43)	**0**.**03**
EGFR CA repeats/overexpression					
LL or LS form/No	66	1		1	
Others	61	1.58 (0.94–2.66)	0.08	1.57 (0.93–2.67)	0.09
SS form/Yes	8	4.11 (1.66–10.14)	**0**.**002**	3.25 (1.33–7.95)	**0**.**01**

HR: hazard ratio; CI: confidence interval. ECS: extra-capsular spread; EGFR CA repeat genotype: S form: <20 repeats; L form: ≥20 repeats.

**Table 5:** Multivariate Cox regression analysis of a combination of *EGFR* CA repeat genotype and protein overexpression among EGFR disomy OSCC patients (n = 135).

	Disease-free survival	*p* value	Overall survival	*p* value
	HR (95% CI)		HR (95% CI)	
Age				
<50 years	1		1	
≥50 years	1.07 (0.64–1.77)	0.80	1.29 (0.77–2.16)	0.33
Differentiation				
Well	1		1	
Moderate/poor	0.98 (0.58–1.66)	0.94	1.29 (0.77–2.18)	0.33
Tumor depth				
<10 mm	1		1	
≥10 mm	2.25 (1.25–4.05)	**0**.**007**	2.44 (1.30–4.58)	**0**.**006**
Nodal status				
(−)metastasis, (−)ECS	1		1	
(+)metastasis, (−)ECS	0.93 (0.42–2.06)	0.85	1.45 (0.71–2.96)	0.31
(+)metastasis, (+)ECS	3.25 (1.79–5.91)	**<0**.**001**	2.85 (1.57–5.17)	**0**.**001**
Primary tumor status				
T1/T2	1		1	
T3/T4	0.69 (0.40–1.19)	0.18	1.17 (0.68–2.02)	0.58
EGFR CA repeats/overexpression				
LL or LS form/No	1		1	
Others	1.25 (0.71–2.19)	0.44	1.41 (0.81–2.46)	0.22
SS form/Yes	2.68 (1.03–6.98)	**0**.**04**	2.41 (0.95–6.15)	0.07

HR: hazard ratio; CI: confidence interval. ECS: extra-capsular spread; DFS: EGFR CA repeats: S form: <20 repeats; L form: ≥20 repeats.

## Discussion

It has been shown that the allelic distribution of the *EGFR* intron 1 CA repeats has interethnic variability^14^ and that this interethnic variability might help to explain the distinct features of *EGFR* amplification and protein overexpression in human cancers among certain populations^21^. The most frequent allele in Asians is the 20 repeat allele, while the 16 repeat allele is the most common among Caucasians. The allele frequencies of the CA repeats observed in this study in terms of the Taiwanese referent controls (52.34% for 20 repeat allele and 19.46% for 16 repeat allele) is in agreement with the previous findings for Asians^14, 21^.

*In vitro*, *EGFR* transcription activity has been found to decline as the number of CA repeats increases and this then correlates with protein expression level *in vivo* ^8^. In addition, a higher number of CA repeats has been found to be correlated with a higher frequency of amplification of the *EGFR* gene in breast cancer cases^21^. In this study, we have observed that a gain of *EGFR* gene copy number can be observed in 30% of the OSCC tumors and this frequency was only slightly increased in tumors from individuals with the CA repeat genotype compared to those with the SS genotype. However, our findings indicated that Taiwanese OSCCs have a significantly higher frequency of *EGFR* amplification compared to German oral cavity cancers (19.6% (38/194) *vs*. 11.5% (24/209)), when analyzed using the same probe and the same amplification criteria^22^. This result is consistent with an interethnic study that consisted of German and Japanese breast cancer cases^21^. Thus, there is clearly an interaction between the number of CA repeats and the frequency of *EGFR* amplification.

The homozygous SS genotype of the *EGFR* intron 1 CA repeats has been found to be associated with an increased risk for glioma, breast cancer and lung cancer^12, 23, 24^. In the present study, we found that individuals with the SS genotype had a significantly higher OSCC risk than those with either of the L form genotypes (OR = 1.65; 95% CI, 1.01–2.70; *p* = 0.05), especially among AQ chewers. In contrast, Kang *et al*. has demonstrated that carriers of >16 CA repeats have a 1.9-fold increased risk of oral cancer among a Puerto Rican population^13^. Conversely, they also found that the risk tended to increase as the number of alleles within the ≥16 CA repeats decreased. These inconsistent findings indicated that cutoff point used to distinguish short and long *EGFR* CA repeat alleles might have a significant effect on the interpretation of any results obtained. One major difficulty of investigating the effects of this polymorphism on protein expression *in vivo* is the wide distribution of CA repeats in terms of number, which leads to many possible heterozygous genotypes. Furthermore, there is no clear model as yet as to how the two alleles interact to give rise to the final phenotype. In these circumstances it is clear that the relevance of this polymorphism to OSCC risk warrants further investigation.

It has been implied that the *EGFR* CA repeats polymorphism might be a potential determinant of protein expression^8, 9^. However, two recent *in vitro* studies have indicated that there is no relationship between EGFR overexpression and the length of the CA repeats present^25, 26^. In addition, EGFR protein overexpression has been attributed to massive gene amplification^25^. Since EGFR protein overexpression, gene copy number and CA repeats have rarely been investigated simultaneously in human primary cancers, the relationship between *EGFR* CA repeats polymorphism and protein expression in human cancers, including head and neck cancer, remains very controversial^10^. In the present analysis, we did not find there to be an association between CA repeats polymorphism and protein expression in OSCC tumors with disomy of the *EGFR* gene. However, it has been demonstrated that there is a significant association between a gain of *EGFR* gene copy number and protein overexpression in Taiwanese OSCC tumors^3, 4^ and thus the influence of the *EGFR* CA repeats polymorphism on protein expression would seem to be minimal in Taiwanese OSCC tumors.

Etienne-Grimaldi *et al*.^9^ reported that EGFR protein expression in head and neck cancer is an independent predictor of specific survival, while CA repeats polymorphism is not an independent predictor of specific survival under the same circumstances. In the present analysis, we found that EGFR protein overexpression and CA repeats was slightly or significantly associated with DFS and OS by univariate analysis. In addition, patients genetically shown to have the *EGFR* CA repeats SS genotype and a tumor with EGFR protein overexpression had a worst prognosis in terms of DFS (*p* = 0.002; HR = 4.11; 95% CI, 1.66–10.14) compared to those patients with the *EGFR* CA repeat LL/LS genotype and/or no EGFR protein overexpression and that this significant relationship still existed (*p* = 0.04; HR = 2.68; 95% CI, 1.03–6.98) after multivariate adjustment for age, primary tumor status, lymph node metastasis, tumor depth, and tumor cell differentiation. Although there was no significant association between *EGFR* CA repeats polymorphism and protein overexpression, these two factors did have a synergistic influence on patients’ prognosis. From the present analysis, it appears that the *EGFR* CA repeat polymorphism may play a role synergistically with tumor EGFR expression level in predicting outcome among OSCC patients. It therefore has significant potential as a biomarker for risk stratification in OSCC. Future studies are needed to confirm our study.

## Electronic supplementary material

Supplementary Table S1 and Figure S1.

Dataset 1

Dataset 2
