# Supplementary material for: Epidermal growth factor receptor intron-1 CA repeat polymorphism on protein expression and clinical outcome in Taiwanese oral squamous cell carcinoma
Source: Sci Rep. 2017 Jul 10;7:4963. doi: 10.1038/s41598-017-04954-5 (PMC5504053; doi:10.1038/s41598-017-04954-5)
Supplement: Supplementary file 1 — Supplementary Table S1 and Figure S1. [file 41598_2017_4954_MOESM1_ESM.pdf]

Epidermal growth factor receptor intron-1 CA repeat polymorphism on protein expression and clinical outcome in Taiwanese oral squamous cell carcinoma

Shiang-Fu Huang[a,b*], Huei-Tzu Chien[b], Wen-Yu Chuang[c], Chih-Hsiung Lai[b], Sou-De Cheng[d], Chun-Ta Liao[a], Hung-Ming Wang[e].

[a]Department of Otolaryngology, Head and Neck Surgery, Chang Gung Memorial Hospital, Tao-Yuan, Taiwan
[b]Department of Public Health, College of Medicine, Chang Gung University, Tao-Yuan, Taiwan
[c]Department of Pathology, Chang Gung Memorial Hospital, Tao-Yuan, Taiwan
[d]Department of Anatomy, College of Medicine, Chang Gung University, Tao-Yuan, Taiwan
[e]Division of Hematology/Oncology, Department of Internal Medicine, Chang Gung Memorial Hospital, Tao-Yuan, Taiwan

*Address for Correspondence: Dr. Shiang-Fu Huang, Department of Otolaryngology, Head and Neck Surgery, Chang Gung Memorial Hospital, Tao-Yuan, 333 Taiwan
E mail: bigmac@adm.cgmh.org.tw

Tel: 886-3-3281200 ext. 3968; Fax: 886-3-3979361.

Supplementary table 1. Characteristics of the OSCC patients (n = 194) and referent controls (n = 1444).

Characteristic	OSCC patients	Referent controls	*p* value
Age (year)			
Mean ± SD	49.34 ± 11.03	45.97 ± 15.97	< 0.001
Range	29-78	15-85	
AQ chewing [N (%)]			
Yes	175 (90.2)	316 (21.9)	< 0.001
No	19 (9.8)	1128 (78.1)	
Cigarette smoking [N (%)]			
Yes	175 (90.2)	830 (57.5)	< 0.001
No	19 (9.8)	614 (42.5)	
Alcohol drinking [N (%)]			
Yes	132 (68.0)	354 (24.5)	< 0.001
No	62 (32.0)	1090 (75.5)	
Site of primary tumor [N (%)]			
Tongue	69 (35.6)		
Bucca	73 (37.6)		
Others	52 (26.8)		
Pathological stage [N (%)]			
Stage I	25 (12.9)		
Stage II	33 (17.0)		
Stage III	32 (16.5)		
Stage IV	104 (53.6)		

SD: standard deviation; AQ: areca quid.

[a] The others of the primary site include mouth floor (n=9), lip (n = 6), alveolar ridge (n = 23), hard palate (n = 4) and retromolar trigone (n = 10).

Figure S1. Distribution of the *EGFR* CA repeats alleles (A) and genotypes (B) in the 1444 referent controls and 194.

Figure S1A

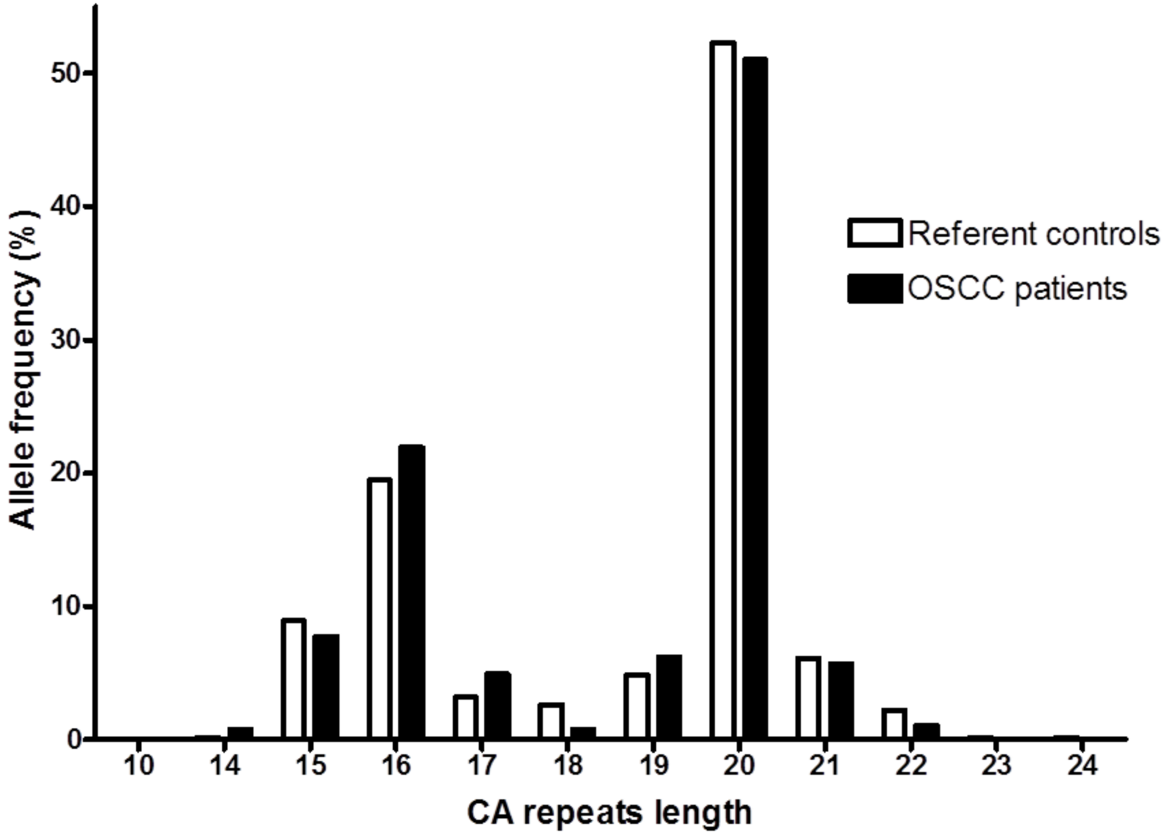

Figure S1B

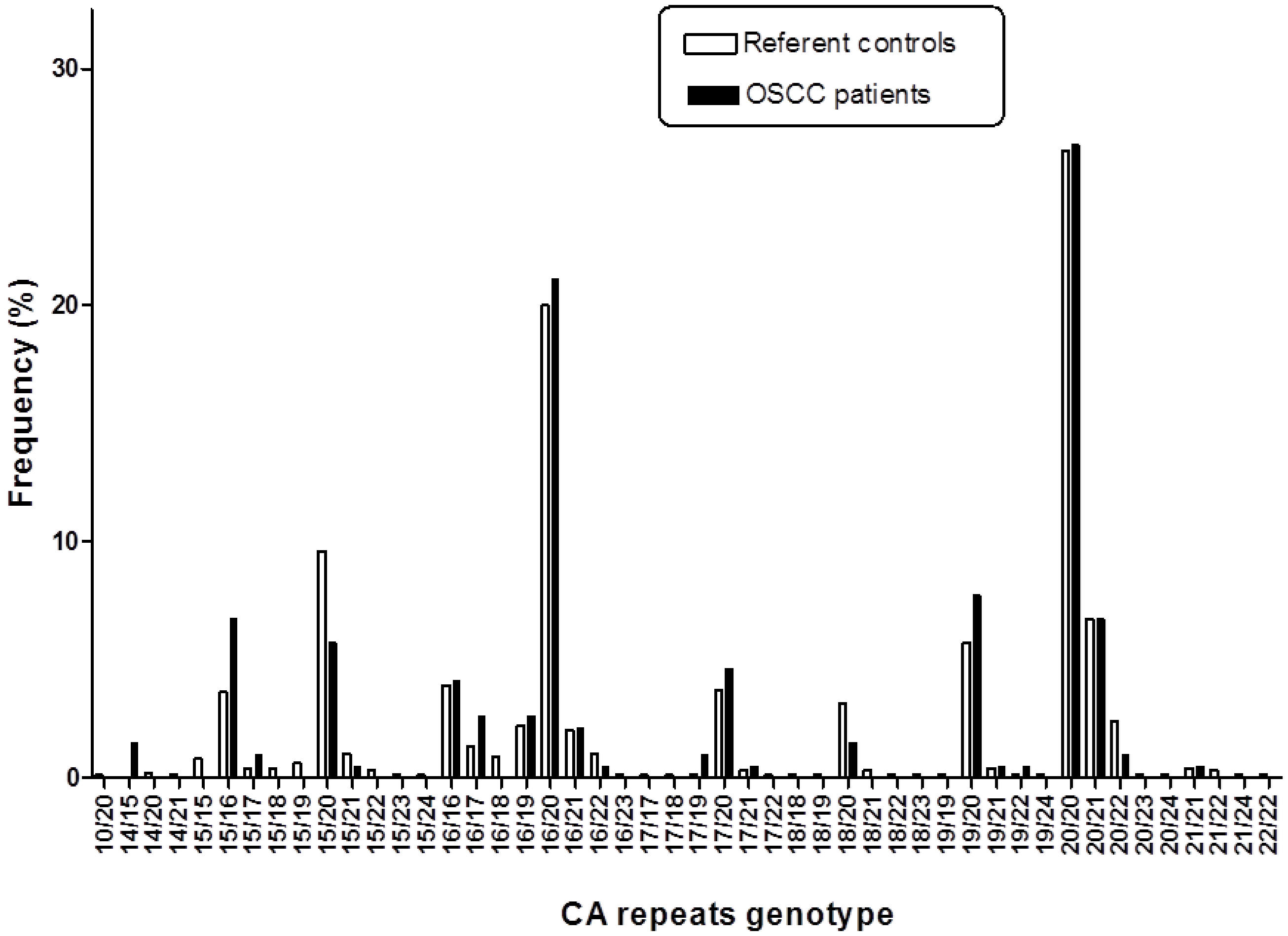
Referent controls
OSCC patients
Frequency (%)
30
20
10
0
10/20
14/15
14/20
14/21
15/15
15/16
15/17
15/18
15/19
15/20
15/21
15/22
15/23
15/24
16/16
16/17
16/18
16/19
16/20
16/21
16/22
16/23
17/17
17/18
17/19
17/20
17/21
17/22
18/18
18/19
18/20
18/21
18/22
18/23
19/19
19/20
19/21
19/22
19/24
20/20
20/21
20/22
20/23
20/24
21/21
21/22
21/24
22/22
CA repeats genotype
